# Experience and Management of Fear in Men's World Cup Alpine Ski Racing

**DOI:** 10.3389/fpsyg.2021.682059

**Published:** 2021-08-02

**Authors:** Morgan Rogers, David Paskevich

**Affiliations:** Faculty of Kinesiology, University of Calgary, Calgary, AB, Canada

**Keywords:** fear, risk-taking, alpine skiing, performance, risk management, instrumental emotion regulation

## Abstract

Alpine ski racers, specifically in the discipline of downhill, may experience fear competing in such a high-risk environment. The purpose of this study was to explore Canadian national team men's experience and management of fear in World Cup alpine ski racing. This study uses an interpretive phenomenological analysis, conducted with five male members of the Canadian national ski team. Three themes emerged: contextual influences, preparation and process, and risk vs. reward. The findings indicate one's experience and management of fear may be influenced by contextual factors (e.g., weather, course profile) and confidence, and that confidence is influenced by the same situational factors that influence fear as well as athlete preparation. There currently exists a discrepancy between the athletes' approaches to training and racing, making it difficult to master fear management strategies. As a result of the discrepancy created between training and racing, there are several implications for how the national team environment and training is structured, and we present recommendations for how these findings can be applied to training.

## Introduction

Olympic and World Cup alpine ski racers all push out of the start gate in pursuit of the same goal: to be the fastest person down the hill, and win the race. Pursuing this goal often involves risk taking and pushing personal limits, which, in the speed discipline of alpine ski racing (i.e., downhill, super-G) means athletes are in very dangerous situations, due to extreme speeds (e.g., exceeding 120 km/h) on steep and icy courses. Risk-taking to achieve performance goals, in an already dangerous situation, results in the potential for elite alpine ski racers to experience fear (ESPNW, 2018).

Smith and Lazarus (1993) conceptualize fear as an emotional response to circumstances where an individual feels threat or danger. Given the highly dangerous nature of alpine ski racing, with the possibility of crashes, injury, and in rare cases death, a World Cup or Olympic ski race can be viewed as a threat to personal safety. Therefore, in line with Smith and Lazarus (1993), who suggest that individuals facing threat to their well-being experience fear, it is possible that alpine ski racers are subject to experiencing fear when competing. Lazarus (1966) suggests that individuals appraise their environment relative to its implication for personal well-being. Appraisal serves to prepare individuals to cope by connecting emotional responses (e.g., fear) with goals (e.g., maximizing benefit or minimizing harm). Lazarus (1966) has outlined two levels of appraisal: (1) primary, where an individual assesses the circumstances relative to personal well-being; and (2) secondary, where an individual assesses their resources and options for coping. Smith and Lazarus (1993) further break down primary appraisal into two components, where an individual assesses whether their emotional response is relevant to their goals, and whether it is congruent with their goals. For an elite alpine ski racer experiencing fear, one might weigh whether their fear is relevant to their goal of skiing fast, and whether it is consistent with their goal of skiing fast.

Instrumental emotion regulation theory suggests that individuals are willing to experience unpleasant emotions (e.g., fear) when the emotion supports goal attainment (Tamir and Ford, 2009). Tamir and Ford (2009), in their virtual simulation experiment with undergraduate students, suggest that fear can be used as an instrumental emotion when engaging in tasks that are threatening. Therefore, an individual's primary appraisal of their fear could be driven in part by individual belief as to whether fear can be used instrumentally (Smith and Lazarus, 1993; Tamir and Ford, 2009). Instrumental emotion regulation has been examined in sport, with Lane et al. (2011) studying instrumental emotion regulation with a survey of 360 runners, finding that 15% of participants felt experiencing anger or anxiety enhanced their performance. In qualitative studies of extreme sports, study participants have shared that when pursuing highly dangerous sports, fear may actually be a necessary emotion, as it ensures a safe course of action (Brymer and Schweitzer, 2012).

While there has been minimal research concerning the experience of fear in competitive athletes, the extreme sport literature (e.g., BASE jumping, big mountain free skiing) provides an understanding of how individuals experience fear in high-risk sports (Slanger and Rudestam, 1997; Brymer and Oades, 2007; Llewellyn et al., 2008; Brymer and Schweitzer, 2012; Buckley, 2016). In the extreme sport literature, fear is typically presented as an emotional response in anticipation of risk-taking behavior. When examining risk-taking in extreme sport settings, athletes' risks are calculated, based on years of experience in the sport and extensive preparation (Brymer and Mackenzie, 2017). In Brymer and Oades' 2007 study of extreme sport athletes, one BASE jumper shared that when deciding to jump, they balanced fear with their knowledge of personal capabilities and technical prowess. Brymer and Mackenzie (2017) further suggested that using the term “risk-taking” may not be appropriate when judging an athlete's choices from the outside. They believe that extreme sport athletes are very well-prepared, highly skilled, and know their limits from years of pushing them, culminating in the athlete's behavior not being viewed as a risk, but rather, a calculated and informed decision.

In Brymer and Schweitzer's 2012 phenomenological study of fear in extreme sport, a participant stated that even with extensive experience, high-risk situations result in fear. However, the participant further stated that fear experienced garners a respect for the situation. This is important to recognize, because it positions fear as a healthy and necessary emotion, with respect for the situation creating awareness for potential dangers and ensuring a safe course of action. Therefore, it is important to recognize that athletes in high-risk sports do experience fear, but it can be managed so that it is facilitative and used to support goal attainment (Brymer and Schweitzer, 2012; Lane et al., 2011). The literature suggests that there are individual differences in how fear is appraised and how an athlete uses unpleasant emotions instrumentally (e.g., fear, anxiety) to facilitate goal attainment. Therefore, the purpose of this study was to explore Canadian national team men's experience and management of fear in World Cup alpine ski racing.

## Methods

### Participants

Participants were five members of the men's Canadian National Alpine Ski Team, who train and compete in the speed disciplines of Downhill and Super-G, and were aged 24 to 37 (*M* = 31.0, SD = 4.95). Participants had 3–16 years of experience on the World Cup Circuit (*M* = 9.8, SD = 5.22), and had all competed at the Olympic games (International Ski Federation, 2019). Participants were selected using a purposive sample, based on the criteria that they (a) were members of the Canadian national team competing in the speed discipline, and (b) had competed on the World Cup Circuit (Smith, 2017). The authors' university ethics board approved this study. All participants provided informed consent prior to partaking in the study.

### Procedure

#### Interview Procedure

The interview guide was 11 questions long, and included broad, open-ended questions such as (a) “What are the signs that indicate you are experiencing fear?” (b) “What are your current strategies to manage fear?” and (c) “How do you experience and manage your arousal before your run compared to during your run?”

A strength of interviewing is the trust and rapport built between participant and researcher (Fontana and Frey, 2000). Therefore, on a separate occasion prior to data collection, the first author attended the Alpine Canada's testing camp to meet participants and develop rapport, as well as outline the study. All participants were given detailed information about the study and an understanding that their data would be kept confidential.

Interviews were then conducted face-to-face by the first author at a training camp later in the year. The pre-season training period was selected for data collection in order to avoid asking questions about fear at or close to a race. Interviews were ~10 minutes in length, providing two to three pages of single-spaced text per interview. While interviews were short in length, the author had previously established a trusting relationship with participants. Additionally, due to the constraints of accessing elite athletes for research (Odendahl and Shaw, 2001), and collecting data at a training camp, the interviews were conducted at the location where a competitor of the participants had died in a training accident 1 year earlier. Therefore, it is possible that the memory of this accident influenced participants' willingness to speak at length on the subject of fear.

#### Data Preparation and Analysis Procedures

Data preparation and analysis followed a series of steps guided by interpretive phenomenological analysis (IPA). First, all interviews were recorded and transcribed verbatim. Transcripts were sent to participants for member checking, and no modifications were made (Creswell and Creswell, 2018). In line with the practice of IPA, analysis began with familiarization of each transcript as a single case. Each transcript was read and re-read, with initial notes and codes identified. These initial codes were then discussed between the two authors, grouped, and translated into themes (Sparkes and Smith, 2013; Smith, 2017).

### Trustworthiness

Several steps were taken to ensure trustworthy data. First, to ensure credibility and confirmability, participants were sent their transcripts, where four participants did not comment, and one confirmed the transcript “looks good!” (Creswell and Creswell, 2018). To ensure further credibility, the second author acted as a critical friend for the first author throughout data analysis, creating conversations that strengthened the overall analysis (Sparkes and Smith, 2013). Finally, to ensure transferability, a thick description was used, which emphasizes using participant quotes to illustrate results (Tracy, 2010).

## Results

We outline three main themes: contextual influences, preparation and process, and risk vs. reward (see Table 1). Subthemes that emerged from contextual influences are: anticipatory anxiety, race day factors, and confidence.

**Table 1:** Results.

**Theme**		**Example**
Contextual influences	Anticipatory anxiety	Fear experienced prior to run, not during
	Race day factors	Weather and snow conditions
		Course profile
		Return from injury
	Confidence	Increased confidence aids in fear management
		Course profile suits athlete's skiing style
Preparation and process		High quality off-season training
		Established pre-competition and competition plans
Risk vs. Reward		Take greater risks in high-caliber races than in training

### Contextual Influences

Certain situations may result in the experience of fear, and racers will not be confident (i.e., a quality that facilitates fear management) in all situations. Fear primarily occurs as an anticipatory emotion prior to pushing out of the start gate, and can be triggered by several situationally specific factors. Conditions and courses can contribute to fear if skiing at a new venue or skiing terrain that doesn't match one's style of skiing. Finally, fear can emerge from situations where athletes have crashed or are returning from injury.

#### Anticipatory Anxiety

When fear is present, it is typically an anticipatory emotion experienced prior to starting a race. Once an athlete begins their run, there may not be room to experience fear. This sentiment can be seen in this statement from one of the skiers:

The hardest part is definitely the arousal before, once you push out of the gate you generally get into a calm because you're able to do what you need to do. I think the hardest part is the anxiety of knowing that you need to do something but not being given the chance to do it just yet, and that gets into your head. So, it's just staying in the moment prior to the race to stay calm so you have more energy and more mental capacity when it's time to do what you need to do. (P5)

All skiers interviewed shared a similar response, that when in the run, the focus is on the present moment. With a focus on the present, and at high speeds, there is typically not room for experiencing fear while in the run, as echoed in the statement “there's not a lot of time to think during the run” (P2). Another skier shared “during the run everything just comes as it is…for me at least it's just instinct most of the time” (P3).

#### Race Day Factors

Conditions and courses are an important variable to consider, because they are an inherent part of the sport, and may incite fear or confidence. Different athletes carry varying skillsets that allow them to excel in certain conditions or on certain courses, and struggle in others. Additionally, when conditions or courses cause uncertainty skiers may experience increased levels of fear. For example, one athlete shared:

Every variable is an added factor, like if you can't see obviously that's going to be scary, if you are unsure of the surface you're pushing your skis into that's another thing that could tack onto the variable of fear. (P2)

Another shared: “Any unknown track you always have a lot more fear at the beginning of that, if you've never gone down something before.” (P3) Based on what emerged, it is evident that doubt may lead to fear, coming either as a result of an athlete's inability to manage courses or conditions that are not favorable, or from uncertainty. Another context specific variable that contributes to fear is an awareness of adverse events, such as watching competitors crash and returning from personal injury. This is reflected in the statement from one participant returning from injury:

For me right now, I'm coming back from an injury, so I've been away from skiing for an entire year and I am struggling with that, it's not, I would say that it is fear actually. When you come back from an injury, your body knows what it wants to do, you know I want to charge…I want to be in an aggressive position, and there's something that just holds you back a little bit…it's just this mental block you have to push through and for some reason after a certain amount of time it releases and then you're able to attack and go after it again. (P4)

#### Confidence

Confidence is context specific, and in the moments where confidence is lacking, fear may be present. For participants, confidence was one of the most frequently cited skills used in fear management. The following statement from one of the interviewees clearly articulates the importance of confidence for fear management: “when the confidence builds, I find the fear or anxiety dissipates and it's replaced by the feeling of being excited.” (P4)

While courses and conditions can be a source of fear by causing uncertainty, they can also be a source of confidence for the athlete when favorable. Several athletes noted that when conditions were “fair” with “good visibility and the snow is consistent all the way down” (P2), it is easy to push and have the confidence to charge. In addition to favorable conditions, certain courses will suit an athlete's skiing style, and if they have success on a particular course they can carry that confidence next time they race that venue.

### Preparation and Process

In the context of this study, preparation is considered all training or tasks undertaken to achieve top performance on race day. Training was the most cited tool related to preparation, and is purposefully scheduled. For example, the Canadian national ski team travels the world during the summer season for on-snow camps. Diverse locations are deliberately chosen in order to train on a variety of courses and snow conditions; the importance of which has been highlighted with the impact of courses and conditions on both confidence and fear. One skier shared this perspective on preparation in relation to fear:

For us it's a not huge thing, I think it's more people looking in from the outside that are scared, like my mom is terrified but it's because she hasn't done the steps to prepare for everything we do. It's normal for us, so the fear is there, even for us looking in, I get more nervous watching other people, then you have to think, they've done everything to prepare as well. (P2)

While preparation is centered on training days, process is focused on race day. Process is the routine that a racer develops and works through each race day and what they focus on during the run. One skier interviewed shared the importance of having a routine because it provides consistency in an environment of constantly changing venues, courses, and conditions.

It helps you to settle into a routine so from there it's just repetitions instead of this is new, today race day, everything's different everything's throwing me off, so I think by having a planned scheduled routine that you practice many times it helps a lot. (P4)

As noted in the contextual influences section, uncertainty may contribute to the experience of fear, and having a routine can significantly minimize uncertainties and aid in arousal management. Grant and Schempp (2013) found that utilizing a pre-competition plan allows athletes to maintain focus on things that are within their control, rather than worrying about factors out of their control. One of the interviewed skiers shares the importance of routine both as a fear management and confidence building strategy:

I think that being prepared in general helps a lot. I have a routine that I try and stick to so if I want to do well on race day the first thing I do in the morning is I wake up and I try to be in a good mood and not eat a lot of energy. Then I go through my routine, I have a plan for when I go free skiing what I want to work on, I have a plan for when I go through inspection, and I think that helps.
How do you get confidence? I mean it's tough to turn it on 1 day and just say I want to be confident, so I think there's a build-up process to that and again I think it has to do with routine, you go out there day after day, you kind of chip away at it, you get better, and as you get better your confidence builds. (P4)

### Risk vs. Reward

Risk vs. reward is also influenced by context. Participants shared they are more inclined to take big risks when there is an equivalent reward, and without an external reward, they may not take risks. For example, all racers stated that they would take their biggest risks at events such as the Olympics, World Championships, or Kitzbühel. Athletes are willing to take bigger risks at these three events because they are the most prestigious events in alpine skiing, and therefore carry the biggest reward for risks taken. It was also unanimous that taking risks in training was not worth it. This collective approach to risk-taking is summarized in the following quotes:

I mean for sure the bigger events I'm more willing to push my limits a lot more, because the reward is bigger. So, it's risk vs. reward a lot of the time. I tend, I as get older especially, I have a harder time to push it in training I find, and I think that's partially because there is no reward for it, other than improving, but it's not like you get to the bottom and there's a medal waiting for you, there's World Cup points, there's prize money—there's none of that. So, it's not worth the risk to take these chances and risk it like maybe a young guy would because he's trying to prove himself to try and get a spot on the team. So, in my age I'm looking more for big events like World Cups or the World Championships. (P4)
Usually the bigger races are when I like to step up and risk everything as much as possible, I really don't like risking anything at all in training…big races like Kitzbühel, World Champs, Olympics—I thrive on how much they're built up. (P1)

As seen in the above quotes, the participants have intentionally selected the moments when they will take risks—based on when matters the most to them. Selectively going all in for the most important races is the epitome of the risk vs. reward attitude, and when the racers decide it's worth it and take that risk, there is no holding back. When interviewees were asked about their own risk management strategies, a common sentiment was “my current risk management strategy would be to risk it all—no safety anymore.” (P5) However, it is important to keep this response grounded in a racing context where risk is on the table. Flørenes et al. (2009) note that in World Cup alpine skiing, the majority of injuries occurred in races, while the least number of injuries occurred in training, which supports this finding that athletes typically take big risks in races. Interestingly, some participants made contradictory statements regarding risk within their interview, which could have implications for how athletes are training and racing, seen in this quote:

Treat it [racing] like every normal day because we do a lot more training than racing and I feel like trying to treat races differently is a recipe for disaster, because they're so far and few between whereas we train tons and tons of days every single year. (P3)

This athlete, like others, had also shared that for any run outside of a World Cup or Championship race, they would not “take any unnecessary risks.” These two quotes highlight an inconsistent philosophy for racing. The risk vs. reward approach is largely based on extrinsic, material rewards and has created a discrepancy between training and racing, which may result in different experiences of fear in the two settings.

## Discussion

The major accordance between this study and the extreme sport literature is that athletes participating in high-risk sports do experience fear, but are able to manage their fear to perform their sport (Brymer and Oades, 2007). However, in contrast to the extreme sport literature, study participants did not speak to using fear to ensure safety or enhance performance. This finding aligns with the work of Lane et al. (2011), who found that while some athletes use unpleasant emotions for performance enhancement the majority (i.e., 85%) do not. While the participants in the present research did not speak the instrumental use of fear, it is still present in elite alpine ski racing. For example, when a Canadian Olympian in alpine skiing retired from competition, he shared with the media that his decision was brought on in part by a teammate's crash and subsequent airlift from the ski hill. His statement to the media demonstrates fear being used instrumentally to make a decision: “[when my teammate] crashed, I thought ‘I should just take the chairlift down.' It took everything for me to push out of the start gate” (Sportsnet, 2018).

In line with self-efficacy theory, which describes situation specific confidence, participants in the present research relied on their confidence to manage their fear (Bandura, 1977). Athletes specifically spoke to using the strongest source of self-efficacy, past performance success, as a means of bolstering their confidence, managing fear, and ultimately taking greater risks to achieve their performance goals. However, it becomes difficult to rely on past performance success to support fear management if the opportunities to experience and manage fear are infrequent, due to the limited risk-taking noted by participants. Therefore, if athletes do not want to push their limits in training, due to the high injury risk, they must find another way to experience and manage fear, in order to boost their confidence in their ability to do so. One potential option for doing this, and a future direction of study, is the use of virtual reality (VR) to train fear management. Nuderscher and Buchheim (2019) note that stress generated with VR is comparable to stress generated *in vivo*, and VR is a promising area for the development of stress management skills.

Coaches and sport psychology practitioners should work with athletes to create an environment that supports safe risk taking, and develops fear management strategies. Building on the preparation section in the results, coaches should continue to thoughtfully plan training so athletes can train on a variety of courses and conditions, train well in the gym to mitigate injury risk (Jordan et al., 2017), and work with ski technicians to ensure athletes have a ski fleet they on confident skiing on (M. Rufener, personal communication, June 1, 2019). Additionally, sport psychology practitioners should work with athletes to help them develop fear management strategies, including self-efficacy, the use of fear as an instrumental emotion, and potentially implementing a VR intervention.

A limitation of the present study is the length of the interviews and limited transferability of results to ski racers of other nations and genders. Future research should conduct interviews in the summer training season at a venue physically removed from ski competition, and should include participants from other nations and genders.

Study results and practical implications are important for use by coaches and support staff of elite alpine skiers when developing a training program. It is important to consider how performance is impacted when athletes train differently than they race, and how training can be structured to be more reflective of racing. Several ways to do so have emerged in this study; such as creating an environment that supports safe risk-taking, and the development of fear management strategies.

## Data Availability Statement

The datasets presented in this article are not readily available in order to protect participant privacy. Requests to access the datasets should be directed to Morgan Rogers, morgan.rogers@ucalgary.ca.

## Ethics Statement

The studies involving human participants were reviewed and approved by University of Calgary Conjoint Health Research Ethics Board. The patients/participants provided their written informed consent to participate in this study.

## Author Contributions

MR and DP conceptualized the study, created the interview guide, and analyzed the data. MR collected the data and wrote the manuscript. DP provided critical revisions and edits on the manuscript. Both authors approved the manuscript in final form.

## Conflict of Interest

The authors declare that the research was conducted in the absence of any commercial or financial relationships that could be construed as a potential conflict of interest.

## Publisher's Note

All claims expressed in this article are solely those of the authors and do not necessarily represent those of their affiliated organizations, or those of the publisher, the editors and the reviewers. Any product that may be evaluated in this article, or claim that may be made by its manufacturer, is not guaranteed or endorsed by the publisher.
